# 7,3′,4′-Trihydroxyisoflavone, a Metabolite of the Soy Isoflavone Daidzein, Suppresses α-Melanocyte-Stimulating Hormone-Induced Melanogenesis by Targeting Melanocortin 1 Receptor

**DOI:** 10.3389/fmolb.2020.577284

**Published:** 2020-12-03

**Authors:** Ji Hye Kim, Jae-Eun Lee, Taewon Kim, Myung Hun Yeom, Jun Seong Park, Eric di Luccio, Hanyong Chen, Zigang Dong, Ki Won Lee, Nam Joo Kang

**Affiliations:** ^1^School of Food Science and Biotechnology, Kyungpook National University, Daegu, South Korea; ^2^Korean Medicine Application Center, Korea Institute of Oriental Medicine, Daegu, South Korea; ^3^Amorepacific Corporation R&D Center, Skin Research Institute, Yongin, South Korea; ^4^Department of Genetic Engineering, School of Life Sciences, College of Natural Sciences, Kyungpook National University, Daegu, South Korea; ^5^The Hormel Institute, University of Minnesota, Austin, MN, United States; ^6^World Class University Biomodulation Major, Department of Agricultural Biotechnology, Seoul National University, Seoul, South Korea; ^7^Advanced Institutes of Convergence Technology, Seoul National University, Suwon, South Korea; ^8^Research Institute of Bio Food Industry, Institute of Green Bio Science and Technology, Seoul National University, Pyeongchang, South Korea

**Keywords:** 7, 3′, 4′-Trihydroxyisoflavone, α-MSH, melanogenesis, depigmentation, melanocortin 1 receptor

## Abstract

7,3′,4′-Trihydroxyisoflavone (7,3′,4′-THIF) is a metabolite of daidzein which is a representative isoflavone found in soybean. Recent studies suggested that 7,3′,4′-THIF exerts a hypopigmentary effect in B16F10 cells, however, its underlying molecular mechanisms and specific target protein remain unknown. Here, we found that 7,3′,4′-THIF, but not daidzein, inhibited α-melanocyte-stimulating hormone (MSH)-induced intracellular and extracellular melanin production in B16F10 cells by directly targeting melanocortin 1 receptor (MC1R). Western blot data showed that 7,3′,4′-THIF inhibited α-MSH-induced tyrosinase, tyrosinase-related protein-1 (TYRP-1), and tyrosinase-related protein-2 (TYRP-2) expressions through the inhibition of Microphthalmia-associated transcription factor (MITF) expression and cAMP response element-binding (CREB) phosphorylation. 7,3′,4′-THIF also inhibited α-MSH-induced dephosphorylation of AKT and phosphorylation of p38 and cAMP-dependent protein kinase (PKA). cAMP and Pull-down assays indicated that 7,3′,4′-THIF strongly inhibited forskolin-induced intracellular cAMP production and bound MC1R directly by competing with α-MSH. Moreover, 7,3′,4′-THIF inhibited α-MSH-induced intracellular melanin production in human epidermal melanocytes (HEMs). Collectively, these results demonstrate that 7,3′,4′-THIF targets MC1R, resulting in the suppression of melanin production, suggesting a protective role for 7,3′,4′-THIF against melanogenesis.

## Introduction

Melanin, synthesized in human melanocytes, plays an important role in protecting the skin from the harmful effects of ultraviolet (UV) radiation (Solano et al., 2006; Park et al., 2009). However, the accumulation of abnormal melanin can cause skin pigmentary disorders such as melasma, freckles and age spots and senile lentigines (Briganti et al., 2003).

The primary cause of hyperpigmentation is known to be exposure of the skin to UV radiation (Park et al., 2009). When exposed to UV radiation, keratinocytes secret α-melanocyte stimulating hormone (MSH). α-MSH stimulates melanocortin 1 receptor (MC1R) and subsequently induces intracellular cAMP production in melanocytes (Solano et al., 2006; Yamaguchi and Hearing, 2009; Gillbro and Olsson, 2011). Elevation of intracellular cAMP stimulates transcriptional factors such as microphthalmia-associated transcription factor (MITF) and cAMP response element-binding protein (CREB) via the cAMP-dependent protein kinase (PKA) and p38 MAPK signaling pathways (Busca and Ballotti, 2000; Smalley and Eisen, 2000; Tada et al., 2002; Saha et al., 2006; Solano et al., 2006; Yamaguchi and Hearing, 2009; Gillbro and Olsson, 2011). In contrast, inhibition of AKT signaling by cAMP blocks MITF degradation (Bellei et al., 2008; Jin et al., 2011). Consequently, the expression of melanogenic enzymes including tyrosinase and tyrosinase-related proteins (TYRPs) is upregulated, resulting in hyperpigmentation (Goding and Fisher, 1997; Sato et al., 1997; Yasumoto et al., 1997). Therefore, suppression of the MC1R signaling pathway may protect strategy against skin hyperpigmentation.

**FIGURE 1:**
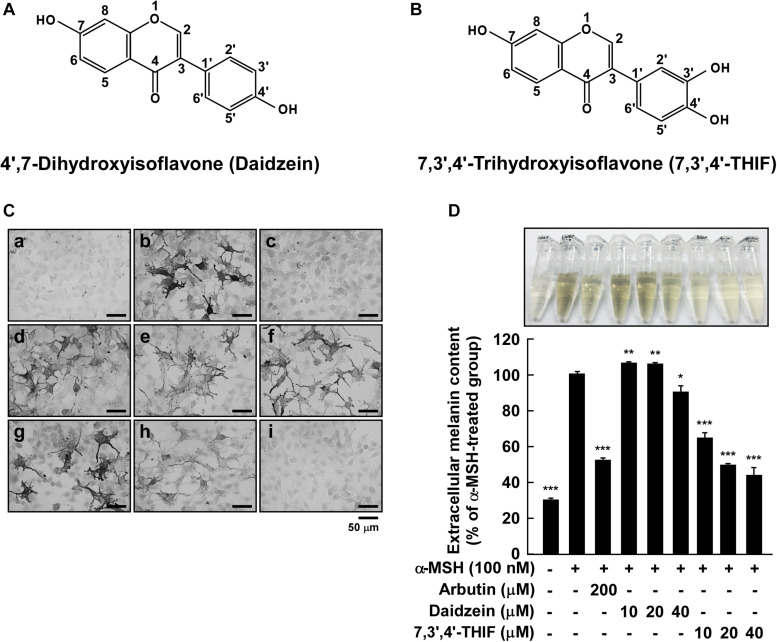
Effect of 7,3′,4′-THIF or daidzein on the α-MSH-induced melanin content in B16F10 melanoma cells. **(A,B)** The chemical structures of daidzein **(A)** and 7,3′,4′-THIF **(B)**. **(C,D)** B16F10 cells were treated as described in “Materials and Methods,” and the melanin contents were measured 3 days later. **(C)** Effects of 7,3′,4′-THIF or daidzein on α-MSH-induced intracellular melanin in untreated control cells (a) and in cells treated with α-MSH (b), α-MSH and 100 μM arbutin (c), α-MSH and 10 μM daidzein (d), α-MSH and 20 μM daidzein (e), α-MSH and 40 μM daidzein (f), α-MSH and 10 μM 7,3′,4′-THIF (g), α-MSH and 20 μM 7,3′,4′-THIF (h), and α-MSH and 40 μM 7,3′,4′-THIF (i). **(D)** Effects of 7,3′,4′-THIF or daidzein on the α-MSH-induced extracellular melanin level in the culture media of B16F10 melanoma cells. The cells were pretreated with samples at the indicated concentrations (10, 20, or 40 μM) for 1 h before being exposed to α-MSH (100 nM) for 3 days. The secreted melanin levels were determined as described in “Materials and Methods.” Asterisks indicate a significant difference (*p < 0.05; **p < 0.01; ***p < 0.001) compared with α-MSH treated groups.

Epidemiologic studies have shown that the dietary consumption of soy may contribute to a reduced the risk of hyperpigmentation and several compounds found in soy have been studied in terms of their ability to promote skin health (Leyden and Wallo, 2011). Previous studies suggested that genistein induces cellular melanin synthesis and enhances tyrosinase activity (Yan et al., 1999). Whereas, daidzein isolated from *Maackia fauriei* inhibited tyrosinase activity, but the effect was weak (Kim et al., 2010). Unlike genistein, daidzein (Figure 1A) is converted to 7,3′,4′-trihydroxyisoflavone (7,3′,4′-THIF or 3′-hydroxydaidzein, Figure 1B) and other compounds in human liver microsomes (Kulling et al., 2001), which can lead to bioactivation. Recently, previous studies have reported that 7,3′,4′-THIF has a depigmenting effect on melanin production (Lee et al., 2006; Goh et al., 2011). Additionally, 7,3′,4′-THIF was shown to inhibit melanin production more effectively than genistein or daidzein in melan-a cells (Park et al., 2010). However, the underlying molecular mechanisms and specific target protein of 7,3′,4′-THIF remain unknown. Here, we report that 7,3′,4′-THIF attenuates α-MSH-induced melanogenesis by targeting MC1R.

## Materials and Methods

### Chemicals

7,3′,4′-THIF was purchased from Indofine Chemical Co., Inc. (Hillsborough, NJ, United States). Dulbecco’s modified Eagle’s medium (DMEM), penicillin-streptomycin and 0.5% trypsin-EDTA were obtained from GIBCO^§^ Invitrogen (Auckland, NZ). Medium 254 and human melanocyte growth supplement (HMGS) purchased from Cascade Biologics (Portland, OR, United States). Fetal bovine serum (FBS), daidzein, α-MSH, arbutin, 3-(4,5-dimethylthiazol-2-yl)-2,5-diphenyltetrazolium bromide (MTT), dimethyl sulfoxide (DMSO), gelatin, forskolin and β-actin antibody were purchased from Sigma Chemical Co. (St. Louis, MO, United States). The antibodies against tyrosinase, tyrosinase-related protein 1 (TYRP-1), tyrosinase-related protein 2 (TYRP-2), MITF, phosphorylated PKA α/β/γ (Thr-198), total PKA, MC1R, goat anti-mouse IgG-HRP, goat anti-rabbit IgG HRP, and donkey anti-goat IgG HRP-conjugated secondary antibodies were purchased from Santa Cruz Biotechnology (Santa Cruz, CA, United States). Antibodies against phosphorylated Akt (Ser-473), phosphorylated mTOR (Ser-2448), phosphorylated GSK-3β (Ser-9), phosphorylated CREB (Ser-133), phosphorylated MKK3/6 (Ser-189/207), phosphorylated MSK1 (Ser-376), total PI3K, total Akt, total mTOR, total GSK-3β, total CREB, total MKK3/6, total MSK1, and total p38 were purchased from Cell Signaling Technology (Danvers, MA, United States). Antibodies against phosphorylated p38 (Tyr-180/Tyr-182) were purchased from BD Biosciences (San Jose, CA, United States). Skim milk was purchased from MB cell (LA, CA, United States). The cAMP immunoassay was purchased from Cayman (Ann Arbor, MI, United States). CNBr-Sepharose 4B and the chemiluminescence detection kit were purchased from Amersham Biosciences (Piscataway, NJ, United States). The protein assay kit was obtained from Bio-Rad Laboratories Inc. (Hercules, CA, United States). Recombinant MC1R was purchased from Millipore (Bedford, MA, United States).

### Cell Culture

The murine melanoma cell line B16F10 was obtained from the Korean Cell Line Bank (Seoul, South Korea). B16F10 cells were cultured in DMEM supplemented with 10% FBS and 1% penicillin/streptomycin at 37°C in a humidified atmosphere with 5% CO_2_. Human epidermal melanocytes (HEMs) derived from moderately pigmented neonatal foreskins were purchased from Cascade Biologics. HEMs were cultured in Medium 254 supplemented with HMGS at 37°C in a humidified atmosphere with 5% CO_2_.

### Cell Viability

B16F10 cells (7 × 10^3^) were cultured in a 96-well plate for 6 h. Cell culture media containing 7,3′,4′-THIF was added at final concentrations of 25, 50, and 100 μM, and then the cells were cultured for 96 h. HEMs (1 × 10^4^) were cultured in a 96-well plate for 6 h. Cell culture media containing 7,3′,4′-THIF was added at final concentrations of 20, 40, and 80 μM, and then the cells were cultured for 72 h. MTT solution (5 mg/mL) were plused 20 μL/well and incubated 2 h. The media were removed and replaced 200 μL of dimethyl sulfoxide (DMSO) per well to dissolve the MTT formazan. After 2 h, absorbance was measured at 570 nm using a microplate reader (Sunrise-Basic Tecan; Grodig, Austria).

### Fontana-Masson Staining

Intracellular melanin accumulation was visualized by Fontana-Masson staining with a slight modification (Joshi et al., 2007; An et al., 2010). Cells were fixed in 100% ethanol for 30 min at room temperature and stained for melanin using a Fontana-Masson staining kit from American Master^∗^Tech Scientific, Inc. (Lodi, CA, United States), according to the manufacturer’s instructions. In brief, cells were stained with ammoniacal silver for 60 min at 60°C, followed by incubation in 0.1% gold chloride and then in 5% sodium thiosulfate. Cell morphology and pigmentation were examined under a Nikon phase-contrast microscope (Tokyo, Japan). The images were analyzed using NIS-Elements 3.0 software.

### Measurement of the Extracellular Melanin Content

The melanin content was measured using a slight modification of a previously reported method (Eisinger and Marko, 1982; Friedmann and Gilchrest, 1987; Gordon et al., 1989). Briefly, cells (8 × 10^3^) were cultured in a six-well plate for 24 h. The culture media was replaced with the media containing daidzein or 7,3′,4′-THIF at the indicated concentrations (10, 20, or 40 μmol L^–1^) for 1 h before being exposed to 100 nmol L^–1^ α-MSH and harvested 3 days later. After treatment, media were collected and the melanin levels therein were determined by measuring the absorbance at 405 nm using an ELISA reader.

### Western Blotting

B16F10 cells (1.5 × 10^4^) were cultured in a 6 cm dish for 48 h then starved in serum-free medium for an additional 24 h to eliminate the influence of FBS on kinase activation. HEMs (9.6 × 10^4^) were cultured in 9 cm dish for 6 days. The culture media was replaced with the media containing 7,3′,4′-THIF (10, 20, or 40 μM) for 1 h before being exposed to 100 nM α-MSH for 3 days. The harvested cells were disrupted and the supernatant fractions were boiled for 5 min. The protein concentration was determined using a dye-binding protein assay kit (Bio-Rad Laboratories Inc.), as described in the manufacturer’s manual. Lysate protein (20–40 μg) was subjected to 10% sodium dodecyl sulfate-polyacrylamide gel electrophoresis (SDS-PAGE) and electrophoretically transferred to a polyvinylidene fluoride membrane (Millipore). After blotting, the membrane was incubated with primary antibodies at 4°C overnight. After hybridization with secondary antibodies, the protein bands were visualized using an ECL plus Western blotting detection system (Amersham^TM^, Piscataway, NJ, United States). The relative intensities were quantified by Image J program.

### cAMP Immunoassay

cAMP levels were measured using a cAMP immunoassay kit (Cayman). Briefly, B16F10 cells were treated with 7,3′,4′-THIF (10, 20, or 40 μM) for 1 h before being exposed to 1 μM forskolin for 30 min. Next, the cells were lysed in 0.1 M HCl to inhibit phosphodiesterase activity. The supernatants were then collected, neutralized, and diluted. After neutralization and dilution, a fixed amount of cAMP conjugate was added to compete with cAMP in the cell lysate for sites on rabbit polyclonal antibodies immobilized on a 96-well plate. After washing to remove excess conjugated and unbound cAMP, substrate solution was added to the wells to determine the activity of the bound enzyme. The color development was then stopped, after which the absorbance was read at 415 nm. The intensity of the color was inversely proportional to the cAMP concentration in the cell lysate.

### *In vitro* and *ex vivo* Pull-Down Assays

Recombinant MC1R (25 μg) or a B16F10 cellular supernatant fraction (1,000 μg) was incubated with 7,3′,4′-THIF-Sepharose 4B beads (or Sepharose 4B alone as a control) (100 μL, 50% slurry) in reaction buffer [50 mM Tris (pH 7.5), 5 mM EDTA, 150 mM NaCl, 1 mM DTT, 0.01% Non-idet P-40, 2 μg/mL bovine serum albumin, 0.02 mM PMSF, and 1 × protease inhibitor mixture]. After incubation with gentle rocking overnight at 4°C, the beads were washed five times with buffer [50 mM Tris (pH 7.5), 5 mM EDTA, 150 mM NaCl, 1 mM DTT, 0.01% Non-idet P-40, and 0.02 mM PMSF], and the proteins bound to the beads were analyzed by immunoblotting.

### α-MSH and 7,3′,4′-THIF Competition Assay

B16F10 cellular supernatant fraction (1,000 μg) was incubated with 100 μL of 7,3′,4′-THIF-Sepharose 4B beads or 100 μL of Sepharose 4B in a reaction buffer (see *in vitro* and *ex vivo* pull-down assays) for 24 h at 4°C, and α-MSH (0.1, 1, 10, or 100 μM) was added to a final volume of 500 μL and incubated for 24 h. The samples were washed, and proteins were then detected by western blotting.

### Molecular Modeling and Energy Minimization

To investigate the detailed molecular basis of MC1R inhibition by 7,3′,4′-THIF, modeling study with 7,3′,4′-THIF and MC1R was performed twice. First, the sequence based search showed that MC1R has a similarity of 47% with a MC4R theoretical model (PDB entry: 2IQP) (Pogozheva et al., 2005), then the MC1R structure was built by using Prime v3.2 from Schrödinger suite 2013 (Jacobson et al., 2002, 2004) based this MC4R theoretical model, the loops of the MC1R homology structure were refined and minimized for docking studying. 7,3′,4′-THIF and daidzein were prepared under LigPrep with default parameter. The grid for docking was generated based on the binding sites that were predicted by SiteMap, finally, Flexible docking was performed in the extra precision (XP) mode. The number of poses per ligand was set to 10 in the post-docking minimization. Second, the sequence regions for the catalytic domains in human MC1R were identified using the PFAM profile database. Unfortunately, no experimental structure exists for the human MC1R. Therefore, models of the catalytic domains in human MC1R were built by homology modeling using closest templates available in the protein data bank (PDB). A BLAST search against the PDB identified the X-ray crystal structures 1CJK and 1AB8 with 67.8 and 38.8% sequence identity, respectively. After a careful multiple-sequence alignment with ClustalW V2.1, 100 models were generated with Modeller V9.10 (Sali and Blundell, 1993; Thompson et al., 1994). The modeled regions were 1-282 (MC1R). The best model, according to the intrinsic Modeller DOPE function, was chosen for docking studies (Krivov et al., 2009). Stereochemistry was assessed with PROCHECK (Laskowski et al., 1996). The stereochemistry of the best model was manually inspected with COOT (Emsley and Cowtan, 2004). Electrostatics calculations were performed with APBS V1.2.1; the molecular surfaces with the electrostatics properties (blue: positive charges; red: negative charges, with unit + 5/-5 kT/e) were rendered using VMD V1.8.9 and PyMOL V1.5 (Oberoi and Allewell, 1993; Holst et al., 1994; Wallace et al., 1995; Trott and Olson, 2010).

### Docking

Prior to the docking calculation, the missing hydrogen atoms on the receptor were added using PDB2PQR in conjunction with the CHARMm force field (Oberoi and Allewell, 1993). AutoDock Vina was used to perform the dockings (Trott and Olson, 2010). Ligands were prepared with Spartan’10 (Wavefunction, Inc.) and the scripts provided by the AutoDock tools (v1.5.4 r29). The docking grid sizes and positions were set using AutoDock. The grid dimensions were large enough to cover the whole biological unit and centered onto previously defined relevant catalytic and biological areas. The grid size was 26 × 28 × 30 Å (MC1R). In MC1R, the loop region closing the receptor (a.a. 155–163) was set as the flexible region for docking. The lowest docking energy and predicted free binding energy for each compound were retained. These energies were used to sort the VLS results. Top docking solutions were manually inspected using COOT and PyMOL V1.5 to verify and confirm their compatibility with existing knowledge on the receptor (e.g., active site location, binding pockets, and conserved amino acid residues potentially involved in binding).

### Model Analysis and Validation

The models were analyzed using Ligplot (Wallace et al., 1995). Confirmation of the interaction maps was performed manually by visual inspection of the models in COOT (Emsley and Cowtan, 2004).

### Statistical Analysis

Where applicable, the data are expressed as means ± *SD*; Student’s *t*-test was used for single statistical comparisons. A probability value of *p* < 0.05 was used as the criterion for statistical significance.

## Results

### Effect of 7,3′,4′-THIF and Daidzein on the α-MSH-Induced Melanin Content in B16F10 Melanoma Cells

To investigate the whitening effect of 7,3′,4′-THIF and daidzein, we first examined the melanin content in B16F10 melanoma cells using Fontana-Masson staining and a melanin content assay. 7,3′,4′-THIF showed cytotoxicity at 50 μM concentration when treated in B16F10 for 96 h, and 7,3′,4′-THIF were incubated with B16F10 under non-cytotoxic conditions for all experiments (Supplementary Figure S1). Treatment with 7,3′,4′-THIF, but not daidzein, significantly reduced the intracellular melanin content of B16F10 cells in a dose-dependent manner (Figures 1Cg–i). Consistent with this, 7,3′,4′-THIF inhibited the α-MSH-induced extracellular melanin content in a dose-dependent manner (Figure 1D). Additionally, 7,3′,4′-THIF lightened the color of the extracellular culture media to a greater degree than did arbutin, a well-known whitening agent. In contrast, daidzein had no effect on the α-MSH-induced extracellular melanin content at all indicated concentrations (Figure 1D). These results suggest that 7,3′,4′-THIF, but not daidzein, exerts a strong depigmenting effect on B16F10 cells.

### Effect of 7,3′,4′-THIF on the Expression of Melanogenic Enzymes and CREB Phsphorylation

**FIGURE 2:**
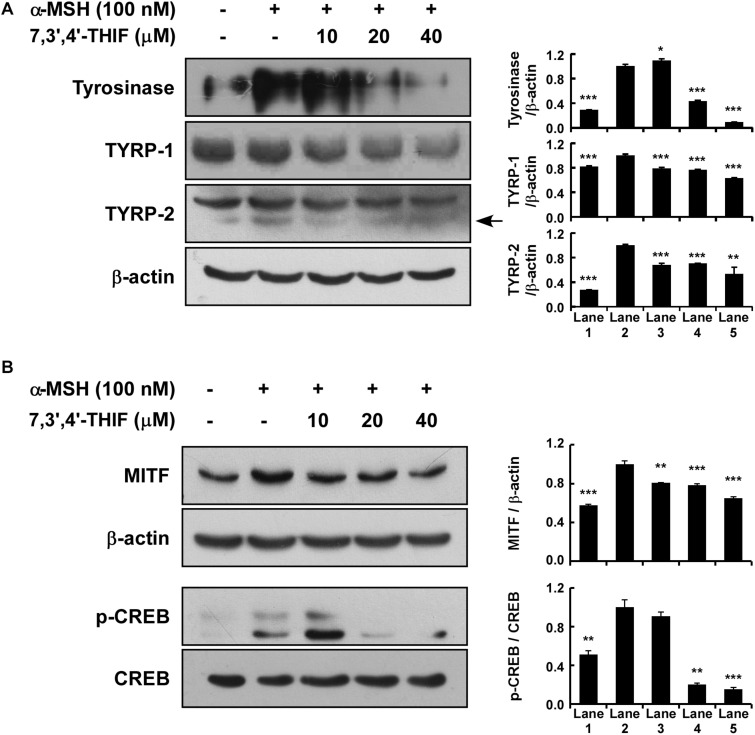
Effect of 7,3′,4′-THIF on melanogenic protein expression in B16F10 melanoma cells. **(A)** 7,3′,4′-THIF suppressed tyrosinase and TYRP-1 but not TYRP-2 expression in B16F10 melanoma cells. To determine the expression of melanogenic enzymes, the cells were pretreated with 7,3′,4′-THIF at the indicated concentrations (10, 20, or 40 μM) for 1 h before being exposed to 100 nM α-MSH for 3 days. **(B)** 7,3′,4′-THIF suppressed MITF transcription factor expression and the phosphorylation of CREB in B16F10 melanoma cells. To examine transcription factor expression, the cells were pretreated with 7,3′,4′-THIF at the indicated concentrations (10, 20, or 40 μM) for 1 h before being exposed to 100 nM α-MSH for 3 days or 30 min. The levels of indicated proteins were determined by Western blot analysis, as described in “Materials and Methods,” using specific antibodies. The data are representative of more than two independent experiments that gave similar results. Asterisks indicate a significant difference (*p < 0.05; **p < 0.01; ***p < 0.001) compared with α-MSH treated group.

Tyrosinase, TYRP-1, and TYRP-2 play important roles in melanogenesis (Park et al., 2009; Yamaguchi and Hearing, 2009; Gillbro and Olsson, 2011). Thus, we examined whether 7,3′,4′-THIF could suppress tyrosinase expression. 7,3′,4′-THIF suppressed α-MSH-induced tyrosinase, TYRP-1 and TYRP-2 expression in B16F10 cells (Figure 2A). Because α-MSH induces tyrosinase and TYRP expression mainly through cAMP/PKA signaling and subsequently induces both MITF expression and CREB phosphorylation, we next investigated whether 7,3′,4′-THIF inhibited MITF expression and CREB phosphorylation. 7,3′,4′-THIF strongly suppressed α-MSH-induced MITF expression and CREB phosphorylation (Figure 2B), suggesting that the whitening effect of 7,3′,4′-THIF is mediated by the suppression of melanogenic protein expression in B16F10 cells.

### Effects of 7,3′,4′-THIF on α-MSH-Mediated Signal Pathways

**FIGURE 3:**
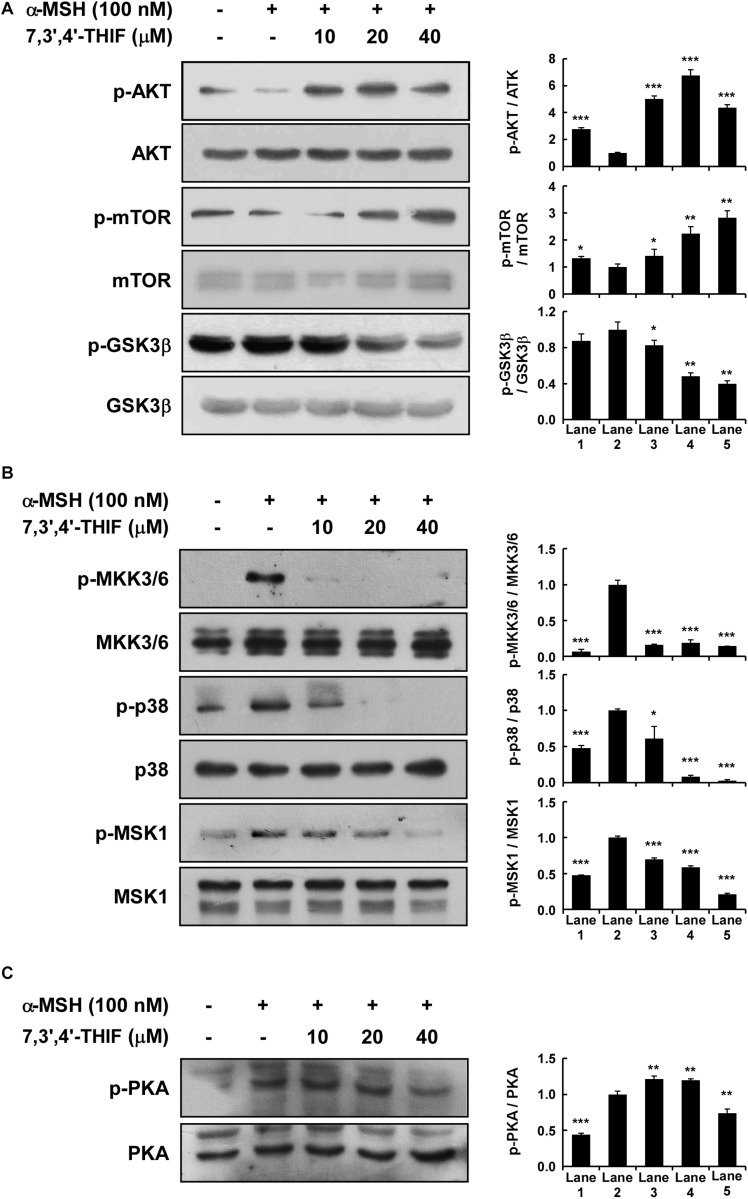
Effect of 7,3′,4′-THIF on the α-MSH-induced dephosphorylation of AKT signaling or phosphorylation of p38 and PKA signaling in B16F10 melanoma cells. **(A)** 7,3′,4′-THIF activated the α-MSH-induced dephosphorylation of AKT. Cells were treated with 7,3′,4′-THIF (10, 20, or 40 μM) for 1 h before being exposed to 100 nM α-MSH and harvested 1 h later. **(B)** 7,3′,4′-THIF inhibited the α-MSH-induced phosphorylation of p38. Cells were treated with 7,3′,4′-THIF (10, 20, or 40 μM) for 1 h before being exposed to 100 nM α-MSH and harvested 15 min later. **(C)** 7,3′,4′-THIF inhibited the α-MSH-induced phosphorylation of PKA. Cells were treated with 7,3′,4′-THIF (10, 20, or 40 μM) for 1 h before being exposed to 100 nM α-MSH and harvested 15 min later. The cells were disrupted and the levels of phosphorylated and total proteins were determined by Western blot analysis, as described in section “Materials and Methods,” using specific antibodies against the respective phosphorylated and total proteins. The data are representative of three independent experiments that gave similar results. The levels of indicated proteins were determined by Western blot analysis, as described in section “Materials and Methods,” using specific antibodies. The data are representative of more than two independent experiments that gave similar results. Asterisks indicate a significant difference (*p < 0.05; **p < 0.01; ***p < 0.001) compared with α-MSH treated group.

Previous studies have shown that AKT, MAPK, and PKA signaling is involved in α-MSH-induced melanogenesis and tyrosinase expression in B16F10 cells (Busca and Ballotti, 2000; Smalley and Eisen, 2000; Tada et al., 2002; Saha et al., 2006; Solano et al., 2006; Bellei et al., 2008; Yamaguchi and Hearing, 2009; Gillbro and Olsson, 2011; Jin et al., 2011). Thus, we next investigated the effect of 7,3′,4′-THIF on AKT, MAPK, and PKA signaling. 7,3′,4′-THIF activated the α-MSH-induced dephosphorylation of AKT, mTOR, and GSK3β in B16F10 cells at the concentrations tested (Figure 3A). 7,3′,4′-THIF also suppressed the α-MSH-induced phosphorylation of MKK3/6, p38, and MSK1 in a dose-dependent manner (Figure 3B). Additionally, 7,3′,4′-THIF suppressed the α-MSH-induced phosphorylation of PKA in B16F10 cells at 40 μM (Figure 3C). Taken together, these results indicate that 7,3′,4′-THIF inhibits tyrosinase expression by activating AKT signaling and suppressing MAPK and PKA signaling.

### Binding of 7,3′,4′-THIF to MC1R

**FIGURE 4:**
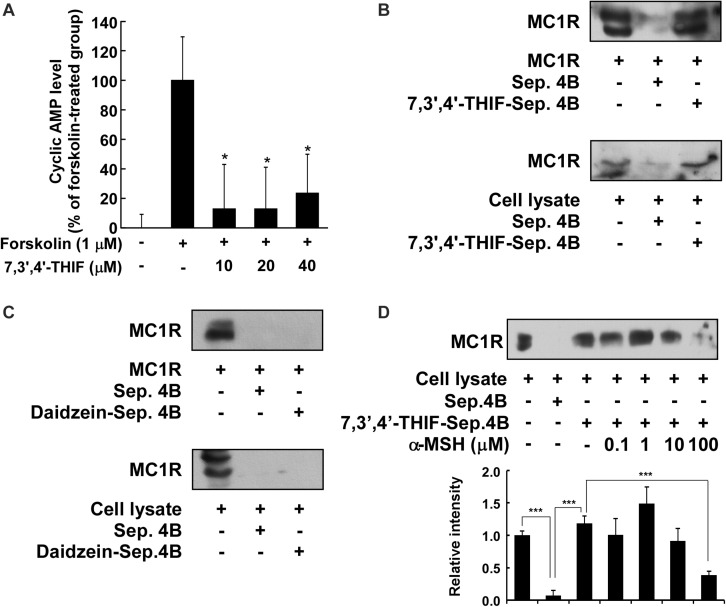
Binding of 7,3′,4′-THIF to MC1R. **(A)** 7,3′,4′-THIF reduced the forskolin-induced cAMP level in B16F10 melanoma cells. The intracellular cAMP level was determined by a cAMP immunoassay as described in section “Materials and Methods.” The results are expressed as the cAMP level relative to the forskolin-treated control. All data are presented as the mean ± *SD* of three independent determinations. Asterisks (*) indicate a significant difference (*p* < 0.05) compared with the forskolin-treated group. **(B)** 7,3′,4′-THIF binds MC1R directly *in vitro* and *ex vivo*. MC1R-7,3′,4′-THIF binding was confirmed by immunoblotting using antibodies against human MC1R (upper panel) or mouse MC1R (lower panel). Lane 1 (input control), human MC1R protein standard or whole B16F10 cell lysates; lane 2 (negative control), Sepharose 4B was used to pull-down MC1R, as described in section “Materials and Methods,” or a B16F10 cell lysate precipitated with Sepharose 4B beads; and lane 3, 7,3′,4′-THIF-Sepharose 4B affinity beads were used to pull-down MC1R or whole B16F10 cell lysates precipitated with 7,3′,4′-THIF-Sepharose 4B affinity beads. **(C)** Daidzein did not bind with MC1R *in vitro* and *ex vivo*. MC1R-daidzein binding was confirmed by immunoblotting using antibodies against human MC1R (upper panel) or mouse MC1R (lower panel). Lane 1 (input control), human MC1R protein standard or whole B16F10 cell lysates; lane 2 (control), Sepharose 4B was used to pull-down MC1R, as described in section “Materials and Methods,” or a B16F10 cell lysate precipitated with Sepharose 4B beads; and lane 3, daidzein-Sepharose 4B affinity beads were used to pull-down MC1R or whole B16F10 cell lysates precipitated with daidzein-Sepharose 4B affinity beads. **(D)** 7,3′,4′-THIF binds to MC1R competitively with α-MSH. B16F10 cellular supernatant fraction (1,000 μg) was incubated with α-MSH at the concentrations indicated (0, 0.1, 1, 10, or 100 μM) and 100 μL of 7,3′,4′-THIF-Sepharose 4B or Sepharose 4B (as a negative control) in a reaction buffer to a final volume of 500 μL. The pulled-down proteins were detected by western blot analysis as described in “Materials and Methods”: lane 1 (input control), whole B16F10 cell lysates; lane 2 (negative control), indicating that neither MC1R binds with Sepharose 4B and lane 3 is the positive control, which indicates that MC1R binds with 7,3′,4′-THIF-Sepharose 4B. Each experiment was performed three times; representative blots are shown. Asterisks indicate a significant difference.

We next investigated whether 7,3′,4′-THIF affected adenylyl cyclase, an upstream effector of the AKT, MAPK, and PKA cascades. After the stimulation of MC1R by α-MSH, adenylyl cyclase converts ATP to cAMP, resulting in the activation of downstream signaling pathways (Solano et al., 2006; Yamaguchi and Hearing, 2009; Gillbro and Olsson, 2011). Therefore, to identify the effect of 7,3′,4′-THIF on the intracellular cAMP level, we performed a cAMP immunoassay using forskolin, a direct activator of adenylyl cyclase. At 40 μM, 7,3′,4′-THIF reduced the forskolin-induced intracellular cAMP level by up to 23.8% (Figure 4A). These results indicate that the inhibition of tyrosinase expression by 7,3′,4′-THIF involves the inhibition of intracellular cAMP formation and adenylyl cyclase activity.

**FIGURE 5:**
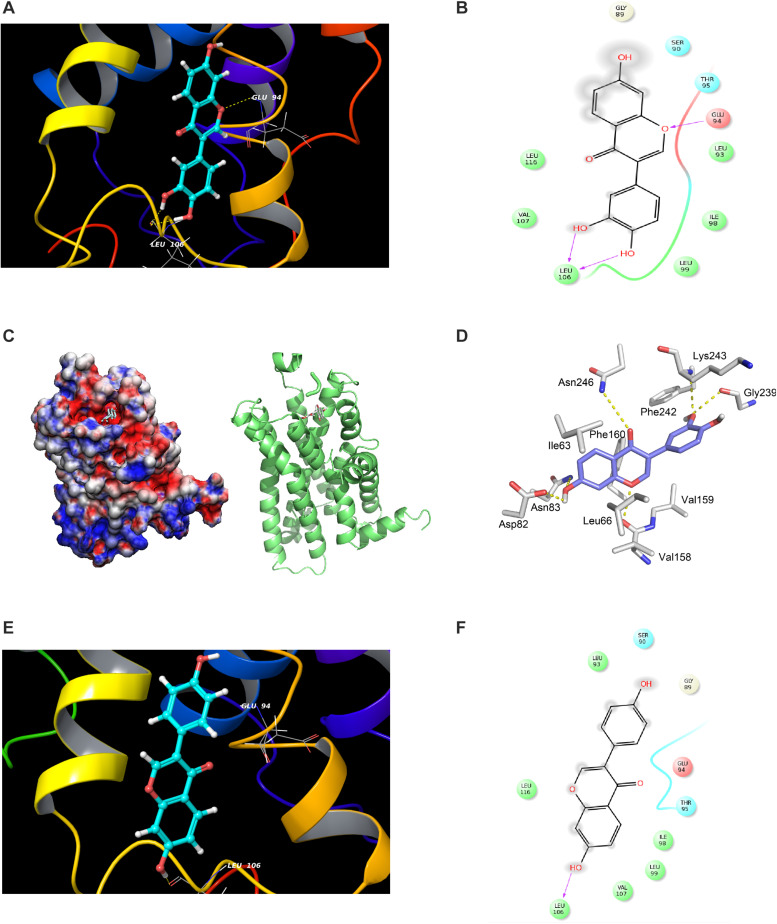
Modeling study of MC1R binding to 7,3′,4′-THIF and daidzein. **(A–D)** 7,3′,4′-THIF binding with MC1R. 7,3′,4′-THIF binding (shown as stick) with MC1R formed hydrogen bonds (yellow dash line) at Glu94 and Leu106 **(A–C)**. Ligand interaction diagram of 7,3′,4′-THIF binding with MC1R **(D)**. The hydroxyl group at the 3’ position of 7,3′,4′-THIF binding with MC1R, formed hydrogen bonds (yellow dash line) with the backbone oxygen of Gly239 and the backbone nitrogen of Lys243 in the MC1R pocket. **(E,F)** Modeling study of MC1R binding to daidzein. Daidzein binding (shown as stick) with MC1R, formed hydrogen bonds (yellow dash line) at Leu106 **(E)** Ligand interaction diagram of daidzein binding with MC1R **(F)**.

Accumulating data suggest that adenylyl cyclase or MC1R is the potential molecular target of 7,3′,4′-THIF, and that binding results in the inhibition of melanogenesis and tyrosinase expression. To confirm whether 7,3′,4′-THIF binds directly to MC1R, we performed an *in vitro* and *ex vivo* pull-down assay using 7,3′,4′-THIF-conjugated and non-conjugated Sepharose 4B beads. MC1R (Figure 4B and Supplementary Figure S2A, lane 3) bound to the 7,3′,4′-THIF-Sepharose 4B beads, but not to the non-conjugated Sepharose-4B beads (Figure 4B and Supplementary Figure S2A, lane 2), revealing that 7,3′,4′-THIF binds MC1R directly *in vitro* and *ex vivo*. However, daidzein did not bind to MC1R both *in vitro* and *ex vivo* (Figure 4C and Supplementary Figure S2B). The binding ability of 7,3′,4′-THIF with MC1R was altered in a concentration-dependent manner in the presence of α-MSH (Figure 4D), suggesting that 7,3′,4′-THIF competes with α-MSH for binding with MC1R. These results suggest that 7,3′,4′-THIF is an α-MSH-competitive inhibitor for suppressing MC1R activation. Computer modeling study also revealed that 7,3′,4′-THIF but not daidzein easily docked to the α-MSH-binding site of MC1R (Figure 5 and Table 1).

**TABLE 1:** Predicted docking energy.

Rank	Docking energy (kcal/mol)	Compound
1	−9.4 (−4.31)	7,3′,4′-Trihydroxyisoflavone (7,3′,4′-THIF)
2	−9.0 (−1.1)	4,7′-Dihydroxyisoflavone (Daidzein)
3	−6.8	α-MSH
4	0.1	Agouti Signaling protein (C-Term)

### Effect of 7,3′,4′-THIF on α-MSH-Induced Melanin Synthesis in HEMs

**FIGURE 6:**
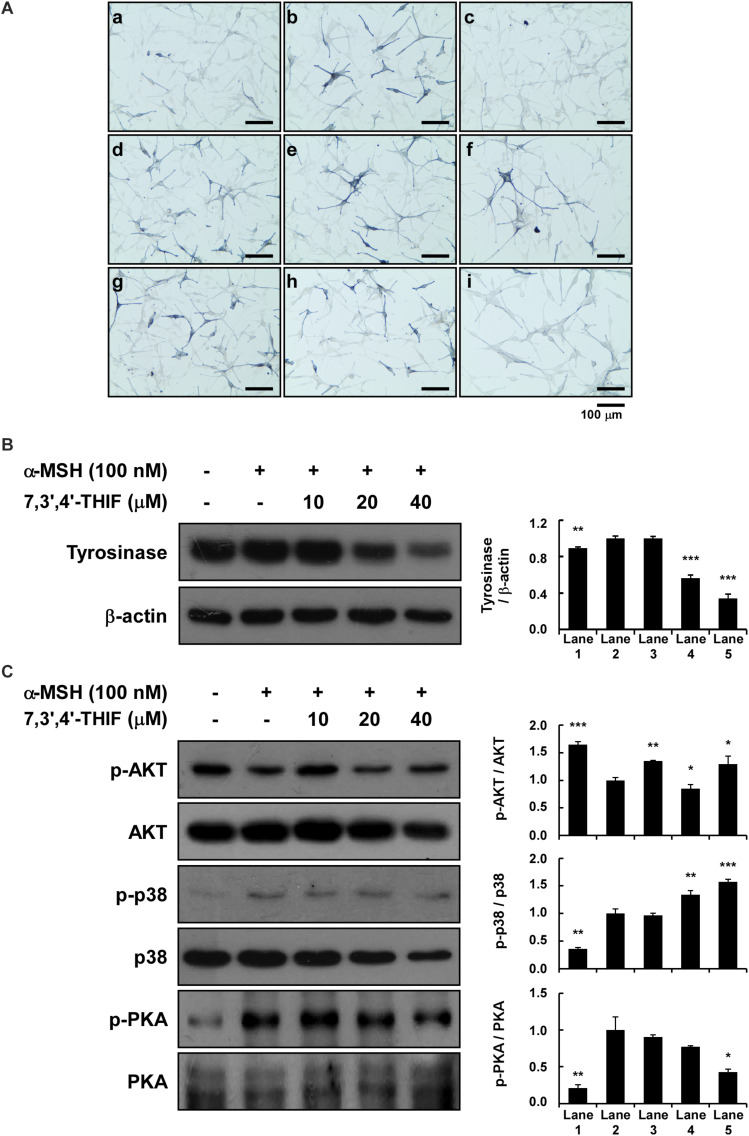
Effect of 7,3′,4′-THIF and daidzein on the α-MSH-induced melanogenesis of HEMs. **(A)** Effects of 7,3′,4′-THIF on α-MSH-induced intracellular melanin in untreated control cells (a) and in cells treated with α-MSH (b), α-MSH and 100 μM arbutin (c), α-MSH and 10 μM daidzein (d), α-MSH and 20 μM daidzein (e), α-MSH and 40 μM daidzein (f), α-MSH and 10 μM 7,3′,4′-THIF (g), α-MSH and 20 μM 7,3′,4′-THIF (h), and α-MSH and 40 μM 7,3′,4′-THIF (i). **(B)** 7,3′,4′-THIF suppressed tyrosinase in HEMs. To determine the expression level, the cells were pretreated with 7,3′,4′-THIF at the indicated concentrations (10, 20, or 40 μM) for 1 h before being exposed to 100 nM α-MSH for 3 days. **(C)** Effect of 7,3′,4′-THIF on the α-MSH-regulated phosphorylation of AKT, p38 and PKA. Cells were treated with 7,3′,4′-THIF (10, 20, or 40 μM) for 1 h before being exposed to 100 nM α-MSH and harvested 0.5 h later. The cells were disrupted and the levels of phosphorylated and total proteins were determined by Western blot analysis, as described in section “Materials and Methods,” using specific antibodies against the respective phosphorylated and total proteins. The data are representative of more than two independent experiments that gave similar results. Asterisks indicate a significant difference (*p < 0.05; **p < 0.01; ***p < 0.001) compared with α-MSH treated group.

We next investigated the whitening effect of 7,3′,4′-THIF and daidzein on HEMs. 7,3′,4′-THIF at a concentration of 80 μM maintained cell viability above 80% for 72 h in HEMs (Supplementary Figure S3). 7,3′,4′-THIF, but not daidzein, significantly reduced the intracellular melanin content of HEMs in a dose-dependent manner (Figure 6A). It was also necessary to verify the mechanisms of 7,3′,4′,-THIF on melanogenesis in the human cells. 20 and 40 μM of 7,3′,4′-THIF significantly reduced α-MSH-induced tyrosinase expression in HEMs (Figure 6B). Furthermore, α-MSH-induced PKA phosphorylation was downregulation in HEMs cultured with 40 μM of 7,3′,4′-THIF, and phosphorylation of Akt inhibited by α-MSH treatment was partially ameliorated by 7,3′,4′-THIF treatment (Figure 6C). 7,3′,4′-THIF also slightly decreased the phosphorylated p38, but the level of p38 also showed a tendency to decrease, showing an increasing phospho-/total- form ratio. These results indicate that 7,3′,4′-THIF could exert the same depigmenting effect in both murine melanoma cells and human melanocytes.

## Discussion

Soybean, one of the most important foods in Asia, is important as a source of protein, as well as an important source of isoflavone (Genovese et al., 2007). In particular, genistein and daidzein are major active compounds of soybeans and their many pharmacological activities have been known. The isoflavones are metabolized in the body, which requires evaluation of physiological activity in the form of their metabolites. 7,3′,4′-THIF is considered one of the main oxidized metabolites of daidzein (Heinonen et al., 2003). Recently, 7,3′,4′-THIF, a metabolite of daidzein, was reported to have beneficial effects on hypopigmentation. Previous studies showed that 7,3′,4′-THIF inhibits melanin production in melan-a (Yan et al., 1999) and B16 melanoma (Goh et al., 2011) cells. Although accumulating evidence suggests that 7,3′,4′-THIF can inhibit hyperpigmentation, the underlying molecular mechanisms and its specific target protein have not been reported yet. Here, we report the marked depigmentation effect of 7,3′,4′-THIF on α-MSH-induced hyperpigmentation and suggest the underlying molecular mechanism and targets.

To determine the effect of 7,3′,4′-THIF on α-MSH-induced melanogenesis, we first investigated its effect on intracellular and extracellular melanin levels in B16F10 melanoma cells. 7,3′,4′-THIF, but not daidzein, inhibited α-MSH-induced intracellular and extracellular melanin production in B16F10 cells. These results indicate that 7,3′,4′-THIF plays a more important role than daidzein in the depigmenting effect of soy.

Previous studies have established the role of tyrosinase and/or TYRPs in B16F10 melanoma cells (Goding and Fisher, 1997; Sato et al., 1997; Yasumoto et al., 1997). Upregulation of the level of several melanogenic enzymes, including tyrosinase and TYRPs, promotes melanin synthesis (Goding and Fisher, 1997; Sato et al., 1997; Yasumoto et al., 1997). Additionally, the expression of tyrosinase is primarily regulated by transcription factors such as MITF and CREB. Therefore, the inhibition of MITF and/or CREB leads to the suppression of melanin synthesis through reduced tyrosinase expression (Goding and Fisher, 1997; Sato et al., 1997; Yasumoto et al., 1997). Our results showed that 7,3′,4′-THIF inhibited the α-MSH-induced tyrosinase, TYRP-1, TYRP-2 and MITF expression and CREB phosphorylation. This transcriptional regulation of tyrosinase by 7,3′,4′-THIF was mediated by the activation of AKT and inhibition of p38 and PKA phosphorylation. 7,3′,4′-THIF also inhibited cAMP production by directly binding with MC1R both *in vitro* and *ex vivo*, competitively with α-MSH. Taken together, these results indicate that the inhibition of tyrosinase, TYRP-1, and TYRP-2 expression by 7,3′,4′-THIF was attributable to the suppression of MC1R activity. The melanocytes for our western blot data had different treatment times of 7,3′,4′-THIF with α-MSH, depending on the proteins to be confirmed. Induction of tyrosinase, TYRPs, and MITF expression required a relatively long treatment of α-MSH for up to 3 days, and a relatively short treatment time (15–60 min) was required to regulate kinase phosphorylation. This is consistent with the sequence of processes in which the signaling pathways regulated by external stimulation regulate the transcriptional activity of MITF, followed by mRNA regulation, and finally the expression of TYR and TYRPs.

Considering the experimental result showing that 7,3′,4′-THIF binds to MC1R, we carried out modeling study to investigate the binding mode of 7,3′,4′-THIF to MC1R. In the previous study, Prusis et al. (1997) suggested that Glu94 of MC1R has great influence on ligand binding and receptor function. Based on our docking model results, only 7,3′,4′-THIF can bind at Glu94 with binding affinities of -4.31 kcal/mol (Figures 5A–D and Table 1), while daidzein can’t, it formed a hydrogen at Leu106 with binding affinities of -1.1 kcal/mol (Figures 5E,F and Table 1). Additionally, in the another model structure of MC1R complexed with 7,3′,4′-THIF, the hydroxyl group at the 3′ position of 7,3′,4′-THIF can potentially form a hydrogen bond with the backbone oxygen of Gly239 and the backbone nitrogen of Lys243 in the MC1R pocket (Figure 5D). Because of the lack of a hydroxyl group at the 3′ position of daidzein, its interaction with the ligand binding site of MC1R would be weaker than that of 7,3′,4′-THIF; thus, daidzein is unable to effectively inhibit MC1R. These results suggested the possible reasons why only 7,3′,4′-THIF can inhibit the activity of MC1R, but not daidzein. Further investigation using X-ray crystallography to determine the structure of the inhibitor complex will elucidate the exact binding modes of 7,3′,4′-THIF with MC1R. Because primary human melanocytes are physiologically relevant to human skin, they have frequently been used for the *in vitro* screening of skin-whitening compounds. Consistent with the above result, 7,3′,4′-THIF significantly reduced intracellular melanin production in HEMs.

**FIGURE 7:**
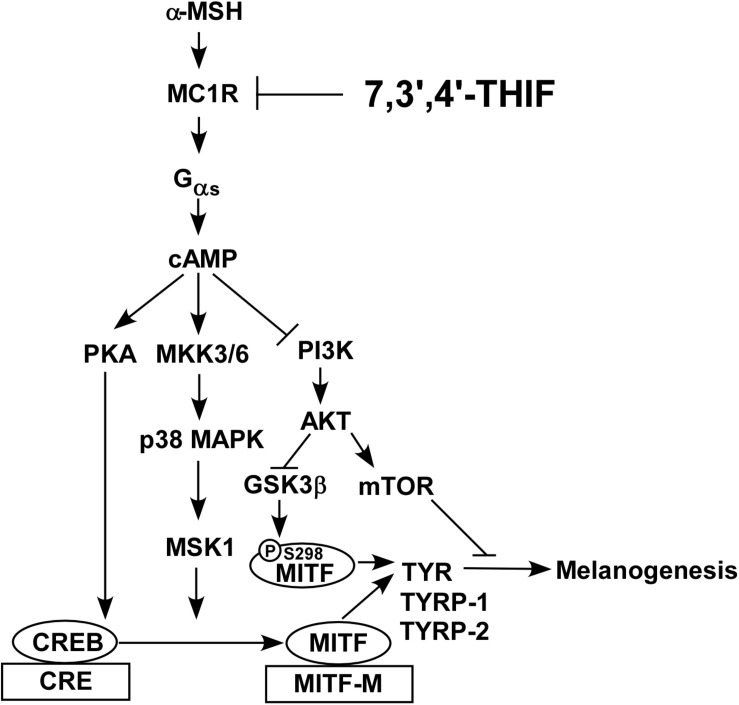
Hypothetical mechanism of the depigmenting activity of 7,3′,4′-THIF.

In summary, 7,3′,4′-THIF, a metabolite of daidzein, more effectively inhibited the α-MSH-induced hyperpigmentation of B16F10 cells than did daidzein. This inhibition is mediated primarily through suppressed MITF expression and CREB phosphorylation, and the subsequent reduced tyrosinase, TYRP-1 and TYRP-2 expressions. 7,3′,4′-THIF inhibited the α-MSH-induced dephosphorylation of AKT and the phosphorylation of p38 and PKA. 7,3′,4′-THIF strongly inhibited the forskolin-induced intracellular cAMP level through binding with MC1R competing with α-MSH. 7,3′,4′-THIF also strongly suppressed α-MSH-induced intracellular melanin production in HEMs. Collectively, these results suggested that MC1R is the potent molecular targets that bind 7,3′,4′-THIF for suppressing hyperpigmentation (Figure 7). These results provide insight into the biological actions of 7,3′,4′-THIF and the molecular basis for the development of new skin whitening agent.

## Data Availability Statement

The datasets presented in this study can be found in online repositories. The names of the repository/repositories and accession number(s) can be found in the article/Supplementary Material.

## Author Contributions

JK and NK contributed to conceptualization of the study. JK, J-EL, EL, HC, and ZD conducted experiment. MY, JP, and KL helped with analyzing the data. JK, J-EL, and NK wrote and edited the manuscript. J-EL, TK, and NK contributed to manuscript revision and approved the submitted version. All authors contributed to the article and approved the submitted version.

## Conflict of Interest

MY and JP were employed by company Amorepacific Corporation R&D Center. The remaining authors declare that the research was conducted in the absence of any commercial or financial relationships that could be construed as a potential conflict of interest.

## Abbreviations

7,3′,4′-THIF: 7,3′,4′-trihydroxyisoflavone
CREB: cAMP response element-binding
DMEM: Dulbecco’s modified Eagle’s medium
DMSO: dimethyl sulfoxide
FBS: fetal bovine serum
HEMs: human epidermal melanocytes
HMGS: human melanocyte growth supplement
MC1R: melanocortin 1 receptor
MITF: microphthalmia-associated transcription factor
PKA: cAMP-dependent protein kinase
TYRP-1: tyrosinase-related potein-1
TYRP-2: tyrosianse-related protein 2
TYRPs: tyrosinase-related proteins
UV: ultraviolet
α-MSH: α-melanocyte -stimulating hormone.
