# Supplementary material for: 7,3′,4′-Trihydroxyisoflavone, a Metabolite of the Soy Isoflavone Daidzein, Suppresses α-Melanocyte-Stimulating Hormone-Induced Melanogenesis by Targeting Melanocortin 1 Receptor
Source: Front Mol Biosci. 2020 Dec 3;7:577284. doi: 10.3389/fmolb.2020.577284 (PMC7747307; doi:10.3389/fmolb.2020.577284)
Supplement: Supplementary file 1 [file Data_Sheet_1.docx]

Supplementary Material

# Supplementary Figures

## Supplementary Figures

**
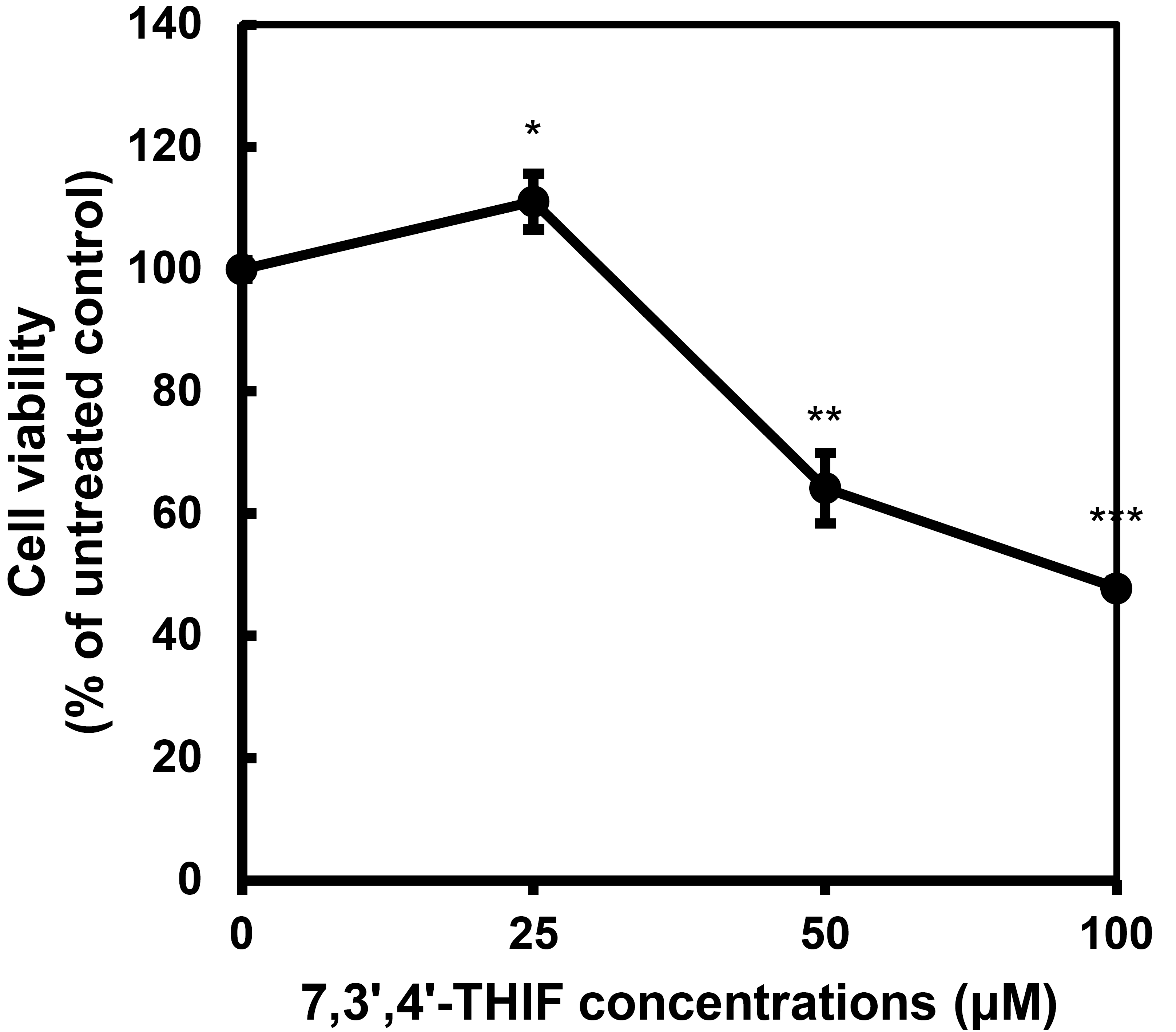
**

**Supplementary Figure 1.** Cytotoxicity of 7,3’,4’-THIF on B16F10. Cells were treated for 96 h and cell viability were determined by MTT assay as described in Material and Methods. Data shown in means ±S.D. (n=3; *, *p*<0.05; **, *p*<0.01; ***, *p*<0.001 vs. untreated control)


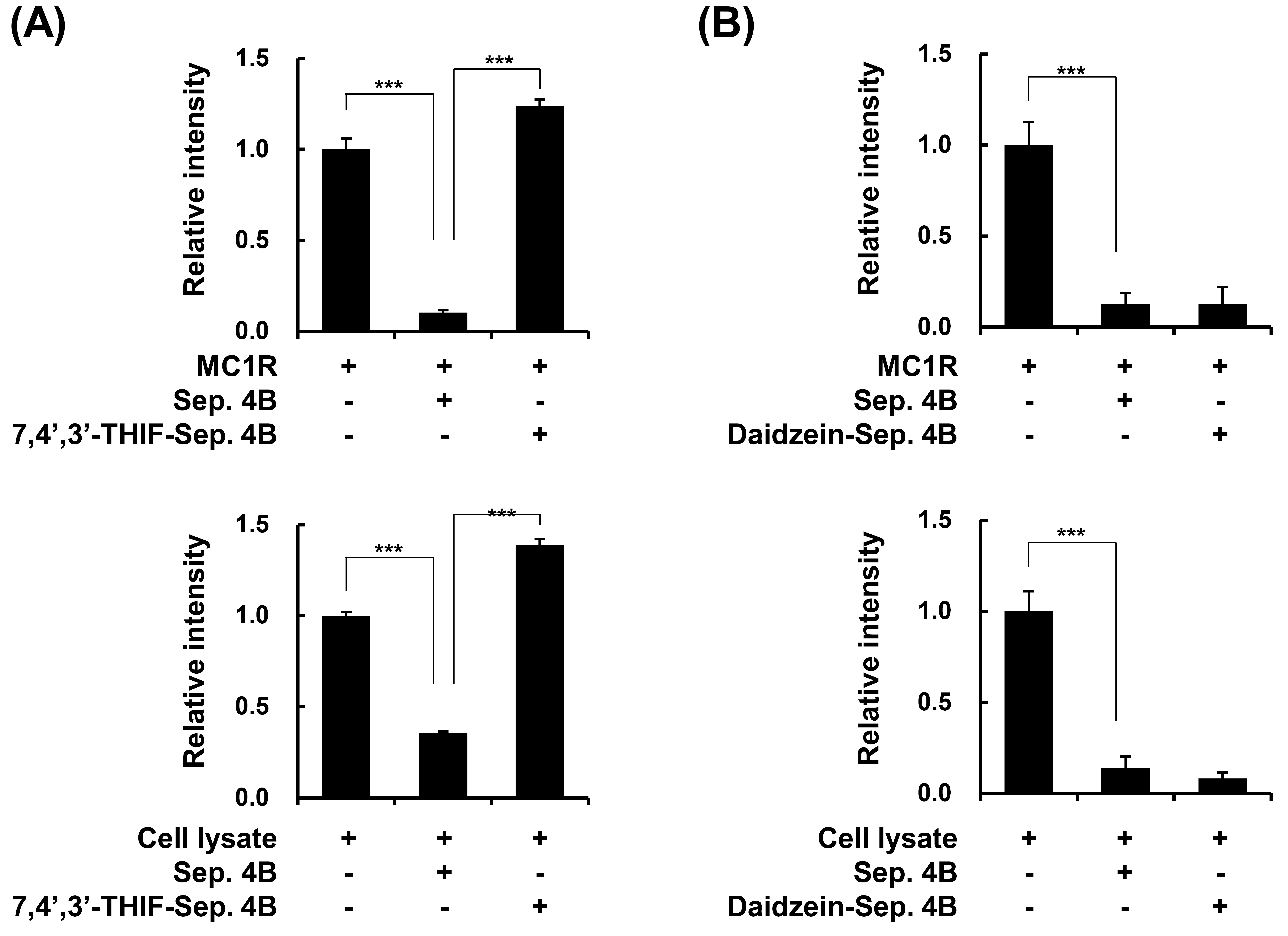


**Supplementary Figure 2.** Binding affinity of 7,3’,4’-THIF and daidzein with MC1R. The data present quantification of MC1R band intensity in Figure 4B (A) and 4C (B). Asterisks indicate a significant difference (*, *p*<0.05; **, *p*<0.01; ***, *p*<0.001).

**Supplementary Figure 3.** Cytotoxicity of 7,3’,4’-THIF on HEMs. Cells were treated for 72 h and cell viability were determined by MTT assay as described in Material and Methods. Data shown in means ±S.D. (n=3; *, *p*<0.05; **, *p*<0.01 vs. untreated control)
